# The Role of Black Pastors in Disseminating COVID-19 Vaccination Information to Black Communities in South Carolina

**DOI:** 10.3390/ijerph19158926

**Published:** 2022-07-22

**Authors:** DaKysha Moore, Lisa N. Mansfield, Elijah O. Onsomu, Nicole Caviness-Ashe

**Affiliations:** 1 Speech Program, John R. and Kathy R. Hairston College of Health and Human Sciences, North Carolina A&T State University, Noble Hall, 1601 East Market Street, Greensboro, NC 27411, USA; 2 Division of General Internal Medicine & Health Services Research, University of California at Los Angeles, 1100 Glendon Ave., Suite 900, Los Angeles, CA 90024, USA; lmansfield@mednet.ucla.edu; 3 Division of Nursing, Winston-Salem State University, 601 S. Martin Luther King Jr. Dr., Winston-Salem, NC 27110, USA; onsomue@wssu.edu; 4 Duke University School of Nursing, Duke University, 307 Trent Drive, Durham, NC 27710, USA; nicole.caviness.ashe@duke.edu

**Keywords:** African Americans, Black churches, communication channels, community, vaccine

## Abstract

COVID-19 vaccination rates have increased since distribution began in December 2020. However, in some states, such as South Carolina, getting people to take the vaccine has been challenging; as of spring 2022, slightly less than 60% of the total population is fully vaccinated. Vaccine hesitancy among Black Americans may be explained by several factors, including lack of confidence in the medical establishment and vaccines in particular. Faith-based leaders, such as pastors, can make a difference. This study explores the communication strategies that pastors in predominantly Black churches use to increase COVID-19 vaccination rates among churchgoers and the surrounding community. We conducted semi-structured interviews with 10 pastors in South Carolina. The main themes that emerged are: (1) using various communication channels to ensure access; (2) representing a trusted source of information; (3) offering a role model for vaccination—leading by example; and (4) strengthening the commitment to health. As the need for COVID-19 vaccination continues, including booster vaccines, pastors can provide accurate information and community outreach to promote the health of Black communities.

## 1. Introduction

During the month of July 2021, more than 26,500 cases of COVID-19 were reported in South Carolina. Those considered “not fully vaccinated” accounted for 88% of positive cases and roughly 80% of deaths [1]. These numbers underscore the importance of getting people vaccinated against COVID-19. However, as of July 2021, the South Carolina Department of Health and Environmental Control (SCDHEC) reported that only 50% of vaccine-eligible state residents had received at least one dose, and “more than a third” of those vaccinated were 65 and older ([2], p. 3). Vaccination rates are even lower among adolescents and young adults, ages 12–24, and public-health efforts are striving to increase them ([3], p. 4). By fall 2021, statistics showed that South Carolina vaccination rates had increased: 61% of eligible residents received at least one dose, and 53% were fully vaccinated (SCDHEC, October 2021).

### The Role of Church Leaders in the Black Community

The church has played an integral role in the Black community, supporting individuals’ spiritual needs and providing a safe space to discuss general concerns. Historically, Black churches have been at the forefront in advocating for social justice [4] and played a pivotal role in advancing the Civil Rights Movement [5]. Its transformative power lies in its leaders. Black pastors have the trust and respect in Black communities needed to facilitate changes in community health behaviors [6,7].

Faith-based leaders, such as pastors of predominantly Black churches, have been called on to help remediate the health disparities in the Black community in South Carolina [8,9]. Pastors have addressed diabetes and emphasize the importance of physical activity [10,11,12] and cancer awareness [13]. Rowland and Isaac-Savage [14,15] surveyed 100 pastors and found that approximately 60% of respondents offered some type of information on health topics, such as diabetes and high blood pressure, to their church members. The authors emphasized the continuing need to explore how Black pastors see their health mission; results could inform more successful health programs in Black communities.

Additionally, more pastors are expressing interest in addressing sensitive health topics, such as HIV/AIDS [16,17,18] and teenage pregnancy [19]. The decision to receive COVID-19 vaccination has been a sensitive and personal topic for many Americans. The disease has taken a disproportionate toll in minority communities across the United States due to disparities in health, and socioeconomics generally; and other factors [20]. Pastors of predominantly Black churches are in a unique position to address the COVID-19 pandemic because of their experiences in tackling various emergencies and reaching out to various community groups [21]. More research is needed to clarify how pastors view their healthcare role to build more successful and sustainable health programs in Black communities. This study explores the communication practices that South Carolina Black pastors used to encourage COVID-19 vaccination among their parishioners.

## 2. Methods

### 2.1. Design and Setting

This study used a phenomenology design to conduct semi-structured telephone interviews with pastors of predominantly Black churches in both rural and urban South Carolina. South Carolina church leaders were chosen, in part, because, at the time of data collection, less than 50% of residents who could be fully vaccinated had decided to get the vaccine [2]. At that time, most of the pastors were using a hybrid model to conduct church services. It included face-to-face meetings indoors and in parking lots and virtual services through social media, such as Facebook or YouTube. Pastors who ministered face-to-face, including outdoor services at different points during the pandemic, also expressed support for adhering to Centers for Disease Control and Prevention (CDC) guidelines to help protect people from contracting the virus.

### 2.2. Sample

We used convenience and snowball sampling methods to identify churches in South Carolina that serve mainly Black residents. Snowball sampling is often used when researchers have difficulty recruiting a specific population [22]. Due to church closures during the COVID-19 pandemic, contacting pastors at the office was difficult. Snowball sampling, in which one participant identifies other potential participants, was the best method for recruitment. The study was approved by the Institutional Review Board before any pastors were contacted.

### 2.3. Recruitment

Recruitment occurred during July 2021. After identifying eligible churches using snowball sampling, one researcher contacted and screened potentially eligible pastors. Once pastors agreed to take part in the study, they were encouraged to provide the names and telephone numbers of other pastors in their network for recruitment. Approximately 14 pastors were contacted through snowball sampling, and 10 agreed to take part in the study. Previous qualitative studies reached data saturation with sample sizes ranging from 8 to 12 participants [23,24]. We reached data saturation following the recruitment of 10 participants.

### 2.4. Interview Guide

The interview guide consisted of 11 questions asking about demographic characteristics, the church’s role in COVID-19 vaccination, the type of church attendance during the pandemic, and channels for disseminating information about the vaccine. Some questions were informed by a previous study on how church leaders communicate information regarding HIV/AIDS [17]. Once verbal consent was given, the audio-recorded interviews took place on the telephone and ranged between 8.5 and 33 min.

### 2.5. Analysis

All interviews were recorded and transcribed verbatim using Otter.ai (Los Altos, CA, USA). The transcriptions were checked for clarity, and the researcher who conducted the interviews and has experience in coding qualitative data completed the task, identifying relevant themes and subthemes. Using open coding, data pertaining to communication strategies addressing COVID-19 vaccines were analyzed in QDA Miner Lite [25]. The main areas coded included: (a) communication channels pastor used to disseminate information on COVID-19 vaccines and testing; (b) offering vaccines and testing sites, and (c) leadership roles. After coding for these three main areas, four distinct themes emerged from the data.

## 3. Results

Ten pastors from predominantly Black churches in South Carolina were enrolled in the study. Most were men (*n* = 7). Half were from the African Methodist Episcopal (AME) denomination (*n* = 5), and the others self-identified as Baptist (*n* = 5). Most of the churches were in large cities, with roughly 100 to 3000 members. Almost all the participants were the head of their respective churches. Four overarching themes were identified to describe the platforms pastors used to communicate COVID-19 vaccine information to church members and surrounding communities: (a) disseminating COVID-19 information through multiple communication channels and accessibility; (b) source of trusted information; (c) a role model for vaccination: lead by example; and (d) strengthening the commitment to health (see Table 1).

### 3.1. Themes

#### 3.1.1. Disseminating COVID-19 Information through Multiple Communication Channels and Accessibility

Pastors reported using different types of communication platforms to share COVID-19 information with church members and the community, including local COVID-19 vaccination clinics and testing centers, and made sure people knew the steps they needed to take to get vaccinated. Pastors described how using various channels, such as social media platforms, enabled them to reach individuals in Black communities who might not have access to accurate and reliable COVID-19 vaccine information. One pastor said:

*So I made sure that our members are getting that information back in, especially when they were testing sites that were testing on a weekly basis. And even with that, [the] vaccine clinic base had available appointments. Anytime that information [would] come down the pipeline, I would shoot that out via text message and also placing announcements in our bulletins each Sunday morning, in the morning or in the parking lot, we will have announcements on it within our program that had announcements on it for a week, and it would include any COVID-19 updates and information*.

(Pastor 3, male, Baptist)

Pastors also maximized the use of interpersonal types of communication, including using church leaders in various ministry groups to disseminate COVID-19 vaccine information. One pastor stated:

*The women’s Missionary Society—and they work hard to try to disseminate information and encourage people to get the vaccine—as well as our class leaders system. And those are like, you know, the congregation is divided into groups, and they serve as leadership over those groups. And they have one-on-one contact, and it’s constantly being said*.

(Pastor 2, female, AME)

Churches were used as COVID-19 vaccination sites to mitigate access barriers affecting Black communities, such as a lack of transportation. Local pastors leveraged existing partnerships with local organizations and medical centers to provide resources for COVID-19 vaccination and to increase access to COVID-19 vaccines. As one pastor described:

*Literally bringing people to vaccination sites … to receive the vaccine—individuals who lived in barrier islands, individuals who live in settlement communities, historic settlement communities. Some Black individuals who would not have had access—they lived in low, outlying areas, and the clinics weren’t necessarily coming to their areas. We use our church buses and transportation*.

(Pastor 7, male, AME)

#### 3.1.2. Source of Trusted Information

Pastors recognized early on that Black communities were skeptical about COVID-19 vaccines and foresaw this skepticism playing a role in COVID-19 vaccination. Pastors acknowledged the communities’ general distrust of the government and believed that “*our people will not get vaccinated … because they ’don’t trust the system’*” (Pastor 2, female, AME). Thus, pastors felt obligated to provide church members and the community with accurate and trustworthy information about COVID-19, emphasizing the health risk that COVID-19 posed for them and their families. The pastors understood and leveraged their trusting relationship with Black communities to address their COVID-19 vaccine concerns and to dispel vaccine misinformation circulating in the community. One pastor said:

*The church is a trusted voice, and so the church’s role is crucial in the sense that, you know, where you see vaccine hesitancy, particularly in the African American community, I mean—who better to help make the case, especially about getting the vaccine in the church? So, you know, I was part of an early campaign—recognizing … that people were hesitant about getting the vaccine—getting it early, when it was my turn to get it, so that people would then be able to see … their leader receiving the vaccine. Nothing happened to me, and so I’m still around*.

(Pastor 7, male, AME)

#### 3.1.3. A Role Model for Vaccination: Lead by Example

In addition to establishing trust between pastors and church members, all pastors emphasized the importance of showing their members that they were willing to take the vaccine. Each pastor interviewed had been immunized for COVID-19. The leaders described the importance of sharing their vaccine experiences with their members and shared photos and videos of themselves getting vaccinated to address the community’s vaccine concerns. As one pastor stated, “*I just tell them about my experience. Tell them, you know, didn’t have any side effects from it. On the second dose that I was feeling, I felt a little sluggish and tired for a couple of days*” (Pastor 3, male, Baptist). Pastors perceived themselves as role models for COVID-19 vaccination and felt that they needed to encourage people to get vaccinated themselves:

*As I teach, I always teach leading by example, you know. If they see a leader stepping up, doing what they’re asking them to do, pretty much will follow suit. And I think that has been the case in our congregation. There’s a lot of our members, you know, that was given the information has gone out to get vaccinated themselves*.

(Pastor 4, male, Baptist)

#### 3.1.4. Strengthening the Commitment to Health

Pastors mentioned the importance of making sure their communities stay as healthy as possible, especially after seeing what COVID-19 has done to the Black community. Pastors reflected on having to preach at funerals of COVID-19 victims, where family members could not attend and say good-bye to loved ones. These personal experiences motivated pastors to strengthen their commitment to being change agents to help reduce the spread of COVID-19 and to “*be more proactive*” in getting their communities’ vaccinated (Pastor 10, male, Baptist).

In strengthening their commitment to health, pastors reported establishing health ministries within their churches to support members through vaccination. Many pastors were motivated to stay up-to-date about COVID-19 to make sure their church members and the surrounding communities were informed about the latest changes in the pandemic and vaccination access and eligibility.

“*We are in the heart as a community. We feel that it is important for the church to speak to all kinds of social, political issues that are in the community, so, frankly, I see myself as a voice in the community to encourage persons to do whatever they can to stem the tide of this pandemic and to prevent themselves from being victims of the latest variant that’s come through*.”

(Pastor 1, male, Baptist)

## 4. Discussion

This qualitative study explored the role and communication strategies that pastors of predominantly Black churches in South Carolina used to increase COVID-19 vaccination rates among churchgoers and the surrounding community. Overall, pastors in this study were committed to improving the health of their church communities and overcoming COVID-19 disparities affecting Black communities. They perceived their role as a trusted source to disseminate relevant and accurate COVID-19 vaccine information and saw themselves as role models to encourage vaccination in Black communities. They used multiple communication channels to increase access to COVID-19 vaccine information and various strategies to reduce structural barriers to COVID-19 vaccination. Similar findings were found in how Black pastors perceived their roles for HIV/AIDS information [17].

Around the globe, COVID-19 has caused many churches to change their community-outreach strategies and to take part in public efforts to promote COVID-19 vaccination. As a study surveyed, most US residents indicated at the beginning of the pandemic that most of their church services were online [26]. They had moved away from face-to-face services and were using social-media platforms for worship.

Pastors reported using multiple communication platforms to disseminate information about COVID-19 vaccines. Churches were in a unique position to reach out to their members and the community when the pandemic forced reliance on online tools. Pastors were using church websites and other media platforms to post information about COVID-19 and how to find local vaccine clinics. One study showed that more Black megachurches are tackling health issues, such as cancer, on their websites [27]. Using various forms of communication to increase COVID-19 vaccination rates is just part of a strategy to encourage more churchgoers and surrounding communities to protect their health.

Pastors viewed themselves as trusted sources to provide accurate and important information about COVID-19 vaccines and felt responsible for making important decisions about church members’ overall health. Harmon et al. [28] noted the importance of trust between Black pastors and their congregations. It allows them to connect and help with their church members’ and the community’s health needs. Pastors emphasized making sure they are open and listen when members express vaccine concerns, without being judgmental about their decisions. In a recent study, Priyor-Dumm and King [29] emphasized leveraging trust in Black pastors as part of an overall strategy for helping organizations reduce COVID-19 vaccine hesitancy. As the push continues to get people vaccinated and receive booster doses, Black pastors can communicate information on the importance of receiving all recommended doses.

The rampant spread of misinformation about COVID-19 and emergent vaccines though social media and various media outlets may have caused some people to become skeptical about COVID-19 vaccination thereby choosing to remain unvaccinated. In Black communities, skepticism about COVID-19 vaccines is heightened by historical and current racism and discrimination in society and medicine and past unethical experimentation such as the Tuskegee syphilis study [30,31,32]. Vaccineaccess barriers also play a role [30,31]. Tasnim, Hossain and Mazumder [33] indicated the importance of utilizing both healthcare providers and community leaders to provide accurate information about COVID-19 to dispel vaccine misinformation. Pastors in this study shared their experiences of COVID-19 vaccination to reduce misinformation and fear about the vaccines and foster trust. Leveraging access to COVID-19 and vaccine information and the social support needed to make decisions may persuade more Black Americans to seek vaccination and the recommended boosters.

As with many other health disparities, COVID-19 has taken an excessive toll on Black communities across the country and in South Carolina [34,35,36]. For this reason, some pastors believed the pandemic reaffirmed their commitment to addressing health disparities. In the past, Black churches have helped to reduce other health conditions disproportionately affecting Black communities [37,38]. Many Black churches are located in rural areas, which tend to have higher COVID-19 mortality rates and lower vaccination rates due to limited access to care and lower educational attainment [39]. Black churches can be used to promote equitable COVID-19 vaccine outreach by increasing access to accurate information and serving as vaccination sites in rural Black communities in South Carolina.

The partnerships the pastors developed with different health agencies and community members to increase COVID-19 vaccination rates showed the importance of working together. A recent study also showed that partnerships between healthcare providers and Black churches might help to close the gap in vaccine disparities among Black and White Americans [40]. Black pastors can provide a physical setting where medical professionals can speak with church members and the surrounding community not only about COVID-19 vaccines, but also about diabetes, obesity, and other health conditions.

The themes that emerged from the study demonstrated that pastors of predominantly Black churches play a crucial role in informing their congregations and communities about COVID-19 and the vaccines. Their dissemination of information about COVID-19 has ranged from interpersonal communication to mass-media strategies. The pastors in the study were also committed to helping people gain access to the vaccine, especially when vaccination sites were limited. However, one of the most important roles the pastors played is as change agents, showing the benefits of being vaccinated themselves. According to Rogers [41], change agents help to form a conduit between access to correct sources of assistance and the client group. In this case, the pastors were facilitating their church members and the surrounding community to receive COVID-19 vaccines. An implication of the study is that Black pastors can connect with local health and other community organizations to increase vaccination rates in South Carolina and should continue to leverage these partnerships for positive health outcomes. Health institutions should consider investing in local Black congregations to support COVID-19 vaccine outreach, including vaccine education trainings to ensure pastors are disseminating accurate and reliable information and resource sharing for COVID-19 testing and vaccination.

This study has several limitations. First, the small sample size and recruitment of pastors from one geographic location limit the generalizability of findings. However, the qualitative design elicited unique insights into communication strategies that pastors of Black churches use to address COVID-19 vaccine information needs of their congregations and communities. Second, the sampling methods used may have limited our ability to identify other pastors of predominantly Black churches who may have provided different perspectives. However, the nonprobability sampling approaches were appropriate given the recruitment limitations, such as church closures during the pandemic. Future quantitative studies are needed to examine which communication strategies among pastors are effective in promoting COVID-19 vaccination.

## 5. Conclusions

As trusted leaders in their communities, Black pastors are in a unique position to promote equitable community outreach for COVID-19 vaccination in Black communities. In this study, pastors played an integral role in increasing access to relevant and accurate COVID-19 vaccine information and reducing structural barriers to vaccine access in Black communities in South Carolina experiencing low vaccination rates. Future public health efforts should support and invest in local Black congregations to establish a sustainable infrastructure to disseminate COVID-19-related information and to provide resources needed for COVID-19 outreach.

## Figures and Tables

**Table 1:** Themes and relevant quotes.

Theme	Relevant Quotes
Disseminating COVID-19 Information through Multiple Communication Channels and Accessibility	“We pretty much utilize all aspects of communication, whether it be Facebook, or Instagram, or in person, bulletins, text message, any way that we can get out information we certainly take, take use of it, or make use of it” (Pastor 4, male, Baptist). “We talk about across the pulpit in online services. I am very hands on with my members, so I speak one on one as well. And, so far, I have not had any members say they don’t want to take the vaccine. All of my members are pretty much so vaccinated except for the young ones as they go by the CDC guidelines as you know some of the little kids cannot vaccinate them yet” (Pastor 5, male, AME). “My church, actually, did the COVID screening three times and the vaccinations twice, and we do have some on hand for those who might change their minds.” (Pastor 6, female, AME) “Every single, every single Sunday? It’s a part of the ongoing announcements, you know, Black churches, we have announcements, right. And, and these things are memorialized for a week, because even after the Sunday broadcast, people are still watching it during the week, on our different platforms, because they’re still there posted. For those who may not have caught Sunday services, they can watch it, you know, after the fact. And you know, that same information is available” (Pastor 7, male, AME). “We use everything that we believe to get the word out [from] our Facebook page, from our website, from YouTube, from radio announcements, from newspaper announcements, within the church from our marque board, from placing signs on the highway. from putting out flyers around the community, and also asking others who have a great social media network to put it on their Facebook page.” (Pastor 8, male, Baptist) “I got the maximum number of followers on my Facebook page. I put it my personal Facebook page. Even now, to this day, I talk about it every Sunday when we live stream” (Pastor 10, male, Baptist).
Source of Trusted Information	“My burden is that our community will keep suffering, because we don’t trust the system and the system doesn’t trust the avenues that we have available to build on. That’s a challenge, and it is a constant concern that our people will not get vaccinated… because they don’t trust the system” (Pastor 2, female, AME). “I think pastors can be a big influence for people to get vaccinated. They have to continue to talk about and encourage people. I am not sure what else we can do. I am a hospice chaplain, and I tell people all the time that COVID is not something that you want to die of. It is not something that you will ever really recover from, so your best bet is to get yourself vaccinated.” (Pastor 9, female, AME)
A Role Model for Vaccination: Lead by Example	“Many of them have express their gratitude that I got vaccinated and posted pictures of it and a video of it and spoke of how it affected me in that second shot. So for all the comments that I’ve received were positive” (Pastor 1, male, Baptist). “I was probably one of the first to be vaccinated twice in February, and then my officers pretty much followed suit—my stewards and trustee and class leaders.” (Pastor 6, female, AME) “We created a little video of not only those of us who are pastors but also leaders in the community, both Black and White. We are talking about police officers and first responders and giving our testimony. We have taken our shot and tried to encourage others to do so as well.” (Pastor 8, male, Baptist) “We partnered with those giving the shots, and we opened our facilities to give the shots. And I acknowledge it and encouraged it. And the greatest acknowledgement that I had was that me and my family had taken it, so I think personally before you can promote it you have to had personally had done it, so we promoted it and we encouraged it. And now, we encourage people to accept the science” (Pastor 10, male, Baptist).
Strengthening the Commitment to Health	“It’s a personal passion of mine. I also serve on the Health Advisory Board for the city, so I get all of the intel, all the information on the demographics that I pastor, of how vulnerable they are to COVID.” (Pastor 2, female, AME) “I told them if I need to go with you to take the shot I will. I have nothing else to do but to pastor my people (Pastor 6, female, AME). “We have to be more proactive and even now I say get the vaccine or get the virus” (Pastor 10, male, Baptist).

## Data Availability

Not applicable.
